# A primer for academic entrepreneurs on academic-industrial partnerships

**DOI:** 10.1038/s41467-021-26103-3

**Published:** 2021-10-01

**Authors:** William N. Hait, Paulus Stoffels

**Affiliations:** 1 Global External Innovation, External Innovation, Johnson & Johnson, 410 Albany Street, New Brunswick, NJ 08933 USA; 2 Vice Chairman Executive Committee and Chief Scientific Officer Executive Committee, Johnson & Johnson, New Brunswick, NJ 08933 USA

**Keywords:** Medical research, Translational research

## Abstract

Partnerships between academic investigators and industry can accelerate the translation of research findings into life-saving products. The healthcare industry has witnessed heightened interest from universities in capitalizing on the discoveries made by faculty to create intellectual property, form new companies and seek investments. However, academic investigators and even Biotechnology start-ups may be unfamiliar with how industry sources and evaluates these opportunities. In this Comment, we share the approaches and principles by which a large healthcare company sources innovation and assesses opportunities to serve as a guide to better deal making with the goal of improving health for humanity.

Interest in translating research discoveries for patient benefit is nothing new. What is newer, however, is the enthusiasm and flexibility within academic institutions for academic entrepreneurship^1^, i.e., for transforming discoveries into life-enhancing, value-creating products by partnering with the healthcare industry. This trend was accelerated by massive increases in investments from public and private sources and attention by regulatory agencies to the need for closer interactions with academic investigators^2^.

Most pharmaceutical companies recognize that a great idea can come from anywhere, not only from within the company’s research laboratories. Industry sources innovation through incubators, partnering offices located within innovation hotspots, (e.g., Boston/Cambridge, South San Francisco, London, Shanghai), and corporate venture funds that both source and fund new projects. The industry also uses a variety of technologies for identifying prospects such as artificial intelligence for trendspotting, i.e., identifying emerging areas of science that have transformative potential. General approaches such as scanning the literature and attending scientific conferences are of some utility, but can often fail to identify high-priority, early opportunities that are not already highly competitive. Finally, investigators approach companies directly to discuss potential collaborations.

The skill sets in industry and academia are complementary. Curiosity-driven research, i.e., the exploration of totally unique areas, is more the purview of academia. In contrast, drug discovery and formulation, regulatory science, manufacturing, and competitive landscaping are the industry’s strengths.

Collaborations between academia and industry can accelerate progress. Companies offer access to internal capabilities (medicinal chemistry, biotechnology resources, toxicology, etc.) to help academic partners (originators) advance their projects. Also, some companies make research space available in incubators where entrepreneurs benefit from cost-efficient access to instruments, reagents, educational programs, and introductions to other investors.

Recently, enthusiasm for investment in biotechnology has substantially increased, fueled by scientific advances, unmet medical needs (e.g., Covid-19), available capital, and initial public offerings (IPOs) including special purpose acquisition companies (SPACS; Box 1). The first quarter of 2021 saw 389 cross-industry IPOs that raised $125 billion^3^.

In this Comment, we provide insights into how our healthcare company evaluates early stage, external opportunities; principles shared by other pharmaceutical companies.

We focus on four essential elements (Table 1). These include: 1. The transformative potential of the idea or asset; 2. the quality and experience of the science and scientific and entrepreneurial leadership team; 3. the ability to define a de-risking, killer experiment and; 4. whether the deal terms are fair to the founder and to the corporation. Finally, opportunities are generally prioritized by corporate strategic areas of interest, internal capabilities, and ones that address substantial unmet medical needs. Ethnic, racial, and gender diversity of the scientific teams and advisory boards add considerable value and are an important consideration.

**Table 1:** The four essential elements of good deal-making.

Element	Description	Considerations
Transformative potential	How likely will the new product exceed the innovation threshold required by the future marketplace	Estimate a ten-year timeframe to market launch
Science and the leadership team	The quality of the science, scientists, and experience, and diversity of the entrepreneurial leadership team	Entrepreneurial experience is often a critical success factor
The killer experiment	The key de-risking experiment	A negative killer experiment does not doom the project. Rather it adds time and money creating opportunity costs
The deal terms	Are they realistic and fair for the originator and the acquirer and in line with similar, recent constructs	Are rights being requested and if so, are they reasonable vs. similar recent terms, i.e., comparables

## Box 1

Glossary of Terms*

**Fair market value**- a reasonable estimate of the selling price of an asset on the open market.

**Initial Public Offering (IPO)**- process of offering shares of a private corporation to the public through a new stock issue.

**Net present value (NPV)**- the expected compound annual return over the life of an investment.

**Non-binding Term Sheet (NBTS)**- an outline of the basic terms and conditions under which an investment may be made.

**Special Purpose Acquisition Company (SPAC)**- a corporation formed for the purpose of raising investment capital through an IPO.

**Large cap**- companies whose stock value is $10 billion or more.

**Pre-money**- how investors value the company today, i.e., prior to an additional investment.

QALY**- Quality-adjusted life year. A measure of the state of health of a person or group in which the benefits, in terms of length of life, are adjusted to reflect the quality of life. One QALY is equal to one year of life in perfect health.

**Small-cap**- companies whose stock value is ~$300 million to $2 billion.

*Definitions modified from Investopedia.

**Modified from NICE.

## Transformative potential

**Fig. 1:**
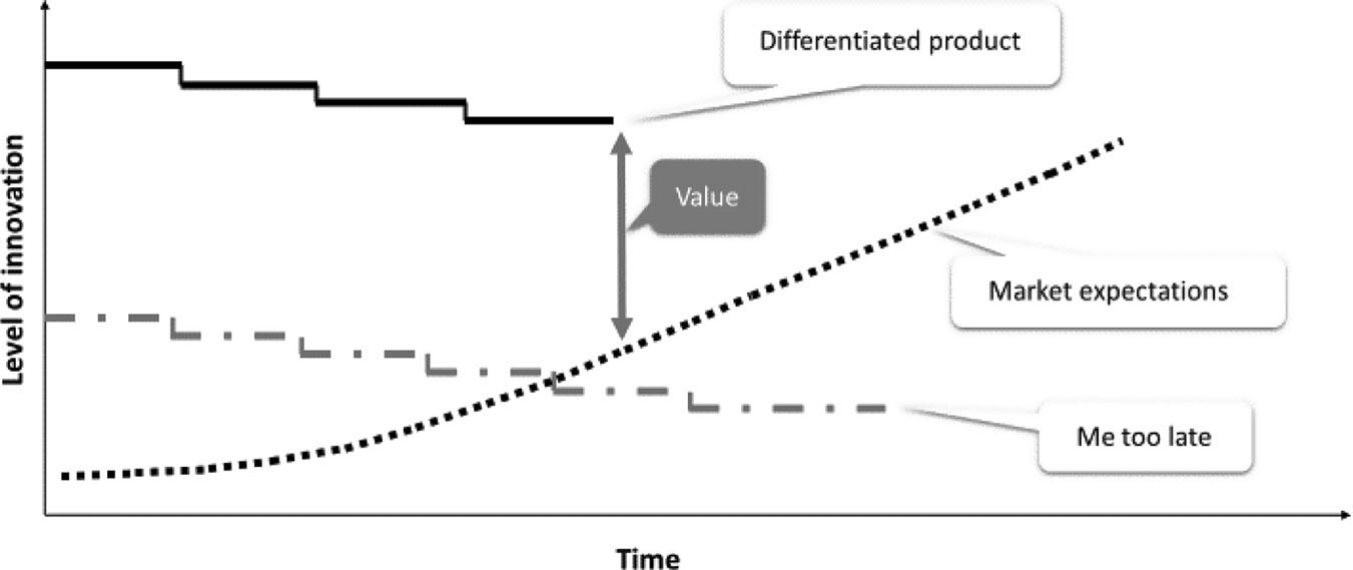
The innovation threshold. Depicts the importance of embarking on a project that will meet market expectations 10 years or more from the time of initiation. The solid back line (top) represents the level of innovation of a differentiated product. The broken gray line (middle) represents the fate of a “me too” drug. The dotted line represents market expectations.

For an idea to ultimately reach the marketplace, it will take time; ten years or more for a new drug with costs that vary widely but can run over $2 billion (when the cost of failures is included)^4^. Therefore, one must consider if ten years from an investment, will the product still be valuable, i.e., will it cross the innovation threshold thereby exceeding market expectations through clinical benefit and commercial potential (Fig. 1)? For example, in the past, the regulatory approval of a new drug with a similar mechanism of action but relatively small improvements in safety and efficacy compared to that of an approved or generic drug might enjoy market success. Today, however, technology assessment agencies such as The National Institute for Health and Care Excellence (NICE) consider whether a new product adds clinical benefit and is cost-effective based on metrics such as gain in quality-adjusted life years (QALYs).

## Science and the scientific leadership team

The ability to drive a product from concept to the market begins with great science, scientific leadership, and data that are reproducible and generalizable. At the early stages of discovery, most data packages are incomplete, but the fundamental dataset must be solid. A company will often ask for a material transfer agreement to independently test an asset. One must also consider whether current technologies exist to advance the project or will new ones need to be created.

The industrialization of an asset is not a skill possessed by most academic scientists, so the academic entrepreneur is wise to deploy individuals with experience working with the Biopharmaceutical industry, i.e., successful serial entrepreneurs. Faculty members may have less experience with large animal toxicology, manufacturability at scale, regulatory agency guidances, and competitive landscapes.

## The killer experiment

In academic labs, a particular problem can be thoroughly investigated for decades through research grants, talented students, and post-docs. In contrast, a drug discovery campaign needs to be rapidly de-risked so that the resources required to move a product forward can be deployed against the most promising projects within a company’s portfolio. A clear de-risking experiment, i.e., a killer experiment, is one designed to disprove the hypothesis underlying the project. For example, if a lead molecule is purported to selectively inhibit a target enzyme in cancer based on biochemical studies against a large panel of enzymes, a killer experiment could be to test if the drug killed cancer cells in cell lines where the target had been deleted. If the killer experiment fails to support the thesis, this does not necessarily mean that given enough time and money that the program will never succeed. Rather, there is an opportunity cost in continuing to pursue this idea rather than investing in others.

## The deal terms

It is difficult to assess the value of an early project. Industry calculates the probability of technical and regulatory success (PTRS) and uses this calculation to approximate the net present value (NPV) of an executed deal. Since early projects may have a negative risk-adjusted net present value (eNPV), an entrepreneur might be disappointed by the terms. Therefore, it is important that deal terms are carefully developed, clearly explained, and are viewed as fair. Important considerations include fair market value determined by analyzing recent, similar types of deal constructs, i.e., comparables, intellectual property filings, and approvals. Companies will want to know the costs to reach the next decision point, for example, the resources required to conduct the killer experiment. Deals will often be constructed with an upfront payment and research support to get to this milestone. The construct will then be backloaded, i.e., payments for later successes, such as the declaration of a clinical candidate, Investigational New Drug (IND) Application or New Drug Application (NDA) filing or approval, the first patient in a Phase 1 clinical study, Phase 2 proof of concept, and entry into a Phase 3 registrational study. Financial terms may also include sales milestones and royalties.

The buyer will then generate a non-binding term sheet (NBTS), which is a preliminary construct of what and how the company would be willing to pay. A NBTS is sometimes required by originators before allowing the potential partner to proceed to a deeper level of due diligence. The NBTS should never be used as a fishing expedition to gain inside or competitive information, but rather it is a good-faith starting point for negotiations.

There are also times when an equity investment in a newly formed company (NewCo) would be beneficial to the partners. Depending on where the company or asset is in its evolution, a valuation is created. It is important for originators to understand the implications of an investment in the context of pre-money and post-money valuations. Pre-money is how investors value the company today, i.e., prior to an additional investment. Post money is the valuation after adding the new investment. For example, if an originator raises $2 million at a $6 million pre-money valuation, the post-money valuation is $8 million. That $2 million invested in a company valued post-money at $8 million gives the investor 25% ownership of the company^5^.

Companies may ask for rights. These include the right of first refusal, i.e., the right to match an offer from a third party, right of first negotiation, i.e., the right to negotiate an offer before the asset is sold to someone else, or a pre-negotiated option price to execute a deal later at an upfront agreed-upon price. If the intellectual property exists, the company may also ask for rights to a sub-license. A company may ask for a period of exclusivity, which has both risks (exclusion of others) and benefits (a dedicated potential buyer) for the originator.

Finally, there are several intangibles. For example, as in any partnership, there should be cultural compatibility. In essence, the chemistry between the originator and the investor can be a deciding factor. Above all, the opportunity for a partnership between academia and industry provides originators with the opportunity to see their work become lifesaving products for millions of people around the world, a shared goal with industry colleagues.

In conclusion, partnerships between academia and industry are essential components of the innovation pipeline, and a better understanding of the criteria used to assess these collaborations can help to streamline the process.
